# Genome wide association study of SNP-, gene-, and pathway-based approaches to identify genes influencing susceptibility to *Staphylococcus aureus* infections

**DOI:** 10.3389/fgene.2014.00125

**Published:** 2014-05-09

**Authors:** Zhan Ye, Daniel A. Vasco, Tonia C. Carter, Murray H. Brilliant, Steven J. Schrodi, Sanjay K. Shukla

**Affiliations:** ^1^Biomedical Informatics Research Center, Marshfield Clinic Research FoundationMarshfield, WI, USA; ^2^Center for Human Genetics, Marshfield Clinic Research FoundationMarshfield, WI, USA

**Keywords:** *Staphylococcus aureus*, skin and soft tissue infection, GWAS, disease susceptibility, integrin and keratin disease pathway

## Abstract

**Background:** We conducted a genome-wide association study (GWAS) to identify specific genetic variants that underlie susceptibility to diseases caused by *Staphylococcus aureus* in humans.

**Methods:** Cases (*n* = 309) and controls (*n* = 2925) were genotyped at 508,921 single nucleotide polymorphisms (SNPs). Cases had at least one laboratory and clinician confirmed disease caused by *S. aureus* whereas controls did not. R-package (for SNP association), EIGENSOFT (to estimate and adjust for population stratification) and gene- (VEGAS) and pathway-based (DAVID, PANTHER, and Ingenuity Pathway Analysis) analyses were performed.

**Results:** No SNP reached genome-wide significance. Four SNPs exceeded the *p* < 10^−5^ threshold including two (rs2455012 and rs7152530) reaching a *p*-value < 10^−7^. The nearby genes were *PDE4B* (rs2455012), *TXNRD2* (rs3804047), *VRK1* and *BCL11B* (rs7152530), and *PNPLA5* (rs470093). The top two findings from the gene-based analysis were *NMRK2* (*p*_*gene*_ = 1.20E-05), which codes an integrin binding molecule (focal adhesion), and *DAPK3* (*p*_*gene*_ = 5.10E-05), a serine/threonine kinase (apoptosis and cytokinesis). The pathway analyses identified epithelial cell responses to mechanical and non-mechanical stress.

**Conclusion:** We identified potential susceptibility genes for *S. aureus* diseases in this preliminary study but confirmation by other studies is needed. The observed associations could be relevant given the complexity of *S. aureus* as a pathogen and its ability to exploit multiple biological pathways to cause infections in humans.

## Introduction

*Staphylococcus aureus* is a complex human pathogen due to its ability to survive both as a carriage organism, and behave as an opportunistic pathogen in a susceptible host. It is a leading cause of invasive bacterial infection that contributes to substantial morbidity and mortality worldwide. This bacterium can cause a variety of diseases ranging from mild to severe skin and soft tissue infections, keratitis, and osteomyelitis to life-threatening bacteremia, pneumonia, endocarditis, and sepsis (Lowy, 1998; Rehm, 2008). Even though *S. aureus* colonizes human anterior nares and other sites on skin in 30–50% of the general population (Graham et al., 2006; Kuehnert et al., 2006; Gordon and Lowy, 2008), not everyone who is colonized gets infected. One reason could be differences in genetic susceptibility to colonization and infections. The established role of host susceptibility in other infectious diseases lends support for a role of host genetics in *S. aureus* infection (de Bakker and Telenti, 2010). Genetic susceptibility to *S. aureus* infections is expected to be complex because this pathogen uses a wide variety of virulence factors that interact with several host pathways to cause disease in humans.

Numerous alleles segregating at a large number of loci contribute to complex disease susceptibility (Yang et al., 2011) with contributions from both common and rare alleles. Because genetic risk factors for *S. aureus* infections have not been previously studied on a genome-wide scale, we utilized the Personalized Medicine Research Project (PMRP) of Marshfield Clinic—a large, population-based biobank of DNA samples—to perform a preliminary genome-wide association study (GWAS) of laboratory-confirmed *S. aureus* infections to discover the underlying host-pathogen interactions.

## Materials and methods

### Subjects

This study utilized Marshfield Clinic's PMRP biobank, a cohort of ~20,000 individuals from 14 Zip Codes surrounding Marshfield, Wisconsin, USA. DNA samples from 3234 PMRP participants were genotyped at >500,000 SNPs as part of the NHGRI/NIGMS-funded eMERGE network (McCarty et al., 2011). These genotypic data are linked to longitudinal electronic medical records (EMR) at the Marshfield Clinic and have served as a powerful resource in previous studies (McCarty et al., 2011; Cross et al., 2012; Hebbring et al., 2013). The population of PMRP is stable and highly homogeneous with a predominant Northern European genetic background and so carries lower risk of confounding by population stratification. All individuals in the study provided informed consent and the study was approved by the Marshfield Clinic IRB.

### Study design

This study examined host susceptibility genes for *S. aureus* infection regardless of *S. aureus* colonization status. We performed a case/control GWAS on a subgroup of the PMRP population: 3234 subjects (309 cases/2925 controls). All study subjects were over 49 years of age. Cases were defined as individuals who had at least one laboratory confirmed test in their medical record of a disease caused by *S. aureus.* Controls were subjects who did not have any evidence of infection due to *S. aureus* in their EMR. We reasoned that because cases had to have evidence of laboratory confirmed *S. aureus* infection in the EMR, patients with no evidence of infection in the EMR would form an ideal control group. The results using our control group are likely very similar to those that would be obtained using population-based controls, as is often employed in GWAS studies (Burton et al., 2007). Only subjects with self-identified Northern European ancestry were included; therefore, both cases and controls had the same genetic ancestry. Additionally, a principal components analysis of their genome-wide genotypes in all study subjects (without knowledge of case/control status) was performed, revealing no evidence of population substructure (Supplemental Figure 1). With this filtered set of case/control subjects, we performed three levels of statistical analysis: SNP-based, Gene-based, and Pathway-based, to identify novel polymorphisms, genes, and pathways involved in susceptibility to *S. aureus* diseases. This hierarchical investigation of genetic effects allows for the incorporation of a gradation of biological information into the statistical tests—the SNP-based scan is the most comprehensive and agnostic to prior biological knowledge, the gene-based analysis uses positional information and collapses effects from the same protein-coding region, and the pathway-based analysis incorporates information obtained from various molecular biology studies.

### Phenotypes

We extracted demographic and medical information on all case/control subjects from the Marshfield Clinic EMR. All cases had an active infection that had yielded *S. aureus* as the major or the only bacterium on a culture plate from a clinical sample such as blood, sputum, etc. Hospital surveillance subjects who were positive for *S. aureus* colonization by PCR were excluded from the study. Age, sex, body mass index (BMI), and Type 2 Diabetes (T2D) status as determined from the EMR were tested for association with case/control status. The binary variables were analyzed using a Fisher's exact test, and the continuous variables were analyzed through a two-tailed *T*-test to test mean differences between cases and controls.

### Genotypic data

The Illumina 660W-QUAD Beadchip array (Illumina, San Diego, California, USA) was used to generate genotype data on over 500 K SNPs from cases and controls. To ease automation of analysis, only data obtained from autosomes were analyzed. After filtering out SNPs with low minor allele frequency (<0.01), missing genotype data (≥0.05 of the study population with missing genotypes) or a significant departure from Hardy-Weinberg equilibrium (HWE) (*p* < 1E-5), there were 508,921 SNPs that passed the quality control screening and were used in subsequent analysis. For a sample to be included in the analysis, we ensured that it had at least 99% of the non-missing SNPs and for each SNP to be included in the analysis, we required that the SNP should have at least 95% of the non-missing subjects.

### Population stratification estimation using EIGENSOFT

We used the EIGENSOFT program (Price et al., 2006) to examine population stratification in our dataset. The program combines population genetics methods and the use of PCA to explicitly capture ancestry differences between cases and controls along continuous axes of variation.

### SNP-based analysis using PLINK and R–Package

PLINK (Purcell et al., 2007), a whole genome association analysis tool set, was used to perform the filtering and QC procedures (as described under “Genotypic data” above) on the raw dataset to generate the data set employed in this study. R is a free software programming language and software environment for statistical computing and graphics (R. Core Team, 2013). The glm() function within R was used to establish the logistic model assuming an additive mode of inheritance with adjustment for the following risk factors: age, gender, BMI, diabetes and the top three principal components estimated from EIGENSOFT for population stratification. The *p*-values calculated from the logistic model were used to test the associations between SNPs and case/control phenotype.

### Gene-based analysis using VEGAS

VEGAS is a software program that tests association between a gene and phenotype trait. VEGAS uses SNP-level data to incorporate information from a full set of markers annotated to each gene and accounts for linkage disequilibrium (LD) between markers (Neale and Sham, 2004; Liu et al., 2010). All SNPs annotated to a gene were used in the calculation, and the method adjusted by the linkage disequilibrium structure by using HapMap data. Monte Carlo simulations from multivariate Gaussian random variables and Cholesky decomposition matrices were employed by VEGAS to produce disease association *p*-values per gene, correcting for the correlation structure between nearby SNPs. This type of analysis has several advantages including the collapsing of effects for all genotyped SNPs within each gene, and reducing the multiple testing burdens. The LD structure of each gene region is factored into the analysis through applying decomposition matrices in the analysis, effectively factoring-out the correlational structure between tightly linked SNPs. Additionally, gene-based results enable the subsequent use of many pathway analysis packages designed to use a single measurement from each gene.

### Pathway-based analysis by DAVID, PANTHER, and ingenuity pathway analysis (IPA) programs

All genes with a *p*-value = 0.01 from the VEGAS analysis were used as input to perform separate pathway analysis by DAVID, PANTHER, and IPA. The DAVID analyses consisted of three steps: measurement of the functional relationship of gene pairs, a DAVID agglomeration procedure to partition genes into functional gene groups, and visualization of the results (Huang et al., 2007, 2009a,b). PANTHER is a publically-available database having gene ontology, functional annotation, and evolutionary conservation information. PANTHER Pathways are available within the database and these pathways were used in our analysis. We calculated *p*-values for enrichment of *S. aureus*-associated genes within specific PANTHER pathways using a standard hypergeometric statistical approach. The statistical over-representation test was implemented in the PANTHER program. A binomial test was used to compare our gene list to a reference list (all human genes) to determine over- or under- representation of genes from our list in PANTHER gene function categories using an experiment-wise approach (experiment-wise α = 0.05) (Mi and Thomas, 2009). Gene-based analyses using IPA (Ingenuity® Systems, www.ingenuity.com, Redwood City, California, USA, www.ingenuity.com) were used to generate protein-protein interaction networks.

## Results

### Population stratification

Using EIGENSOFT to perform PCA of the 508,921 genotypes from all study subjects (without knowledge of case/control status), we found no evidence of strong population stratification (Supplemental Figure 1). Therefore none of the 3234 study subjects were excluded because of population stratification.

### Study subject characteristics

It has been previously noted that male sex, age, high BMI and T2D are risk factors for invasive *S. aureus* infection (Graffunder and Venezia, 2002). As expected, the percent of males was significantly greater in the cases than the control group (51 vs. 39%; *p* = 5.50E-05) (Table 1). There was no significant difference in mean age of case and controls. The prevalence of T2D was significantly higher among cases than controls (22.3 vs. 12.7%; *p* = 1.03E-05) and so was the mean BMI (cases: 32.1 vs. control: 29.5 kg/m^2^; *p* = 3.50E-8). Our findings are consistent with those of previous reports.

**Table 1:** **Demographic and other phenotypic characteristics**.

**Characteristic**	**Cases (N = 309)**	**Controls (N = 2925)**	*p*-value^*^
Age (years) (mean ± *SD*)	74.5 ± 10.9	73.5 (10.8)	0.13
Males (*N*, %)	159 (51.5)	1153 (39.4)	5.50E-05
Type 2 diabetes (N, %)	69 (22.3)	370 (12.7)	1.03E-05
Body mass index (kg/m^2^) (mean ± *SD*)	32.1 ± 8.0	29.5 ± 6.1	3.50E-08

* Two-tailed t-test assuming unequal variances used to compare continuous variables (age and body mass index) and Fisher's exact test used to compare categorical variables (% males and % with type 2 diabetes).

### Q-Q plot

**Figure 1:**
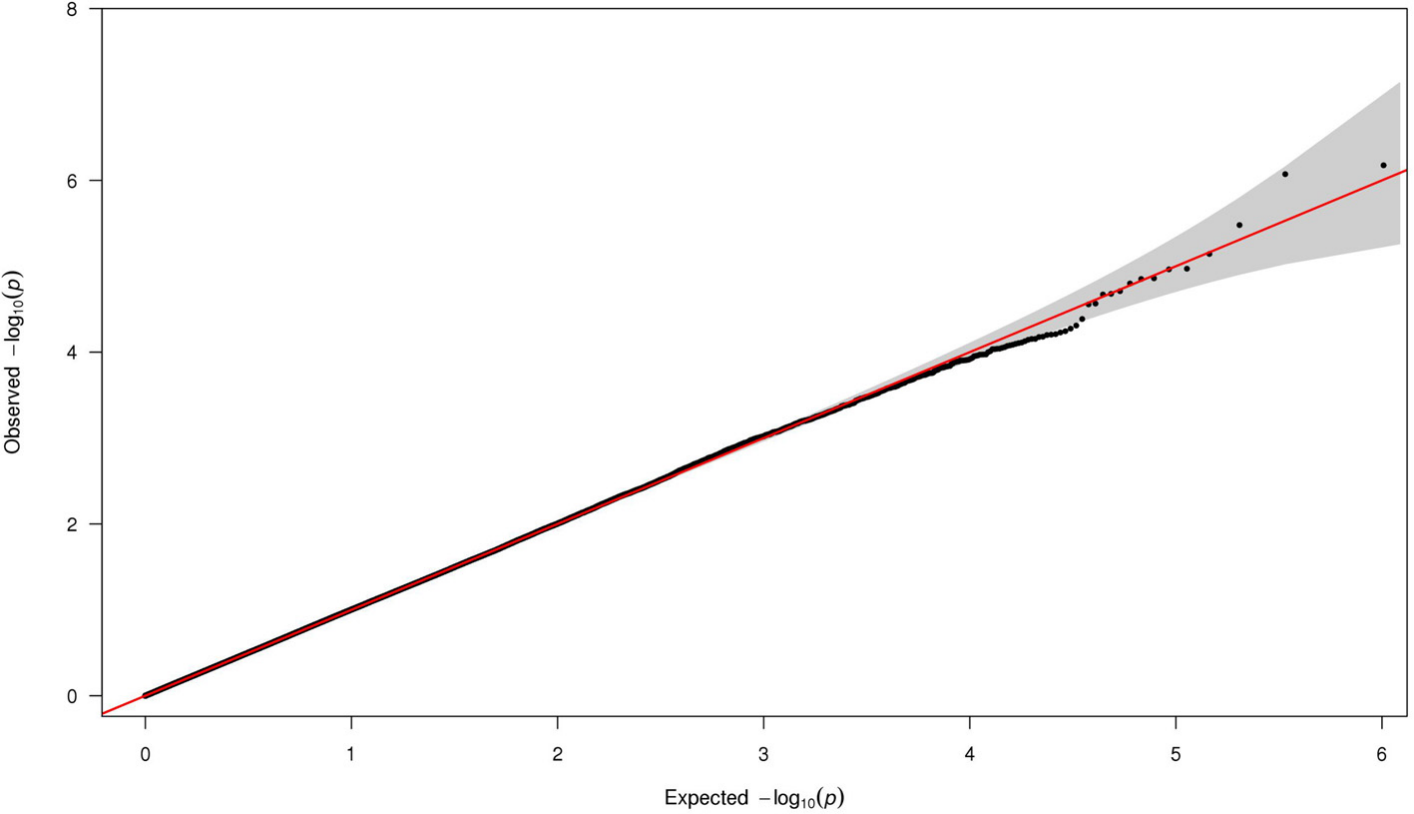
**The Q-Q plot of the *p*-values from all 508,921 SNPs**. The x-axis shows the expected −log_10_(*p*-value). The y-axis shows the observed –log_10_(*p*-value).

We performed Q-Q analysis on the *p*-values obtained using logistic model assuming additive model of inheritance (Figure 1). The plot showed no evidence of population stratification, confounding effects, or systematic bias in the results from the statistical routines employed.

### HWE

Eight hundred forty three SNPs were excluded from the analysis due to departure from HWE exceeding α = 1E-05.

### SNP-based analyses

**Figure 2:**
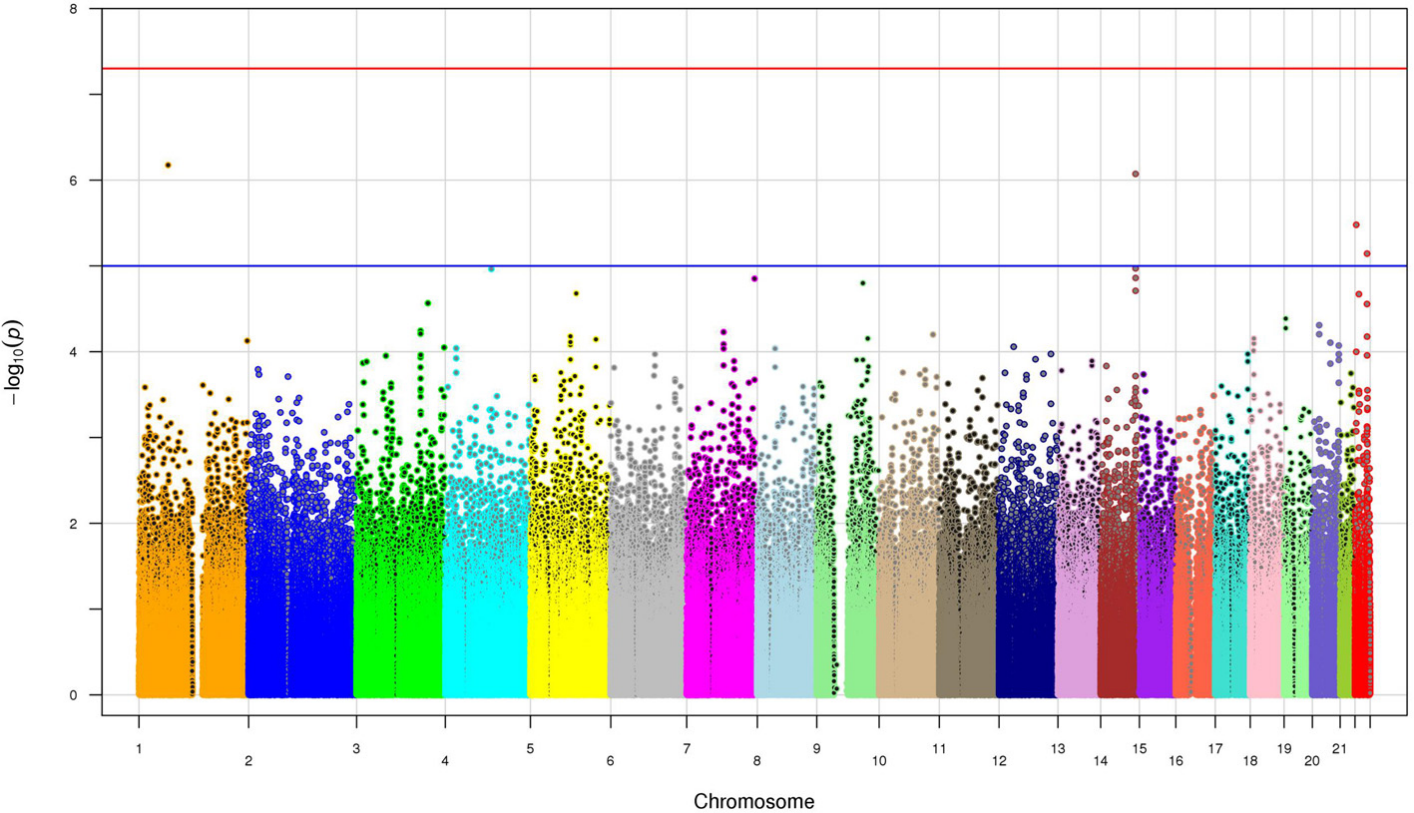
**The manhattan plot of the *p*-values from all 508,921 SNPs**. The x-axis shows the chromosome numbers. The y-axis is the -log_10_ (*p*-value). The blue line is *p*-value of 10^−5^ whereas the red line shows the genome wide significance *p*-value of 5 × 10^−8^.

SNP associations were tested using logistic regression analysis after adjusting for risk factors such as age, gender, BMI, diabetes and three principal components. No single SNP in the GWAS reached the level of genome-wide significance (*p* < 5 × 10^−8^) (Figure 2). However, four SNPs exceeded the *p* < 10^−5^ threshold, including two SNPs (rs2455012 and rs7152530) with a *p* < 10^−7^ (Table 2). Out of these four SNPs, two were intronic (*PDE4B* and *TXNRD2*), one was intergenic with respect to *VRK1* and *BCL11B*, and one was in the 3'UTR of *PNPLA5*. Of the four SNPs on chromosome 14 (Table 2), rs1892234 was in weak linkage disequilibrium (LD) with the other three SNPs (*r*^2^ < 0.42) which were in strong LD with each other (*r*^2^ = 0.80). The two SNPs in *XRN1* were in strong LD (*r*^2^ = 0.92), two of the SNPs on chromosome 22 (rs470093 and rs9614174) were in moderate LD (*r*^2^ = 0.49), and the two SNPs on chromosome 19 exhibited low LD (*r*^2^ = 0.09).

**Table 2:** **Top 20 SNPs, their chromosomal locations, associated genes, major and minor alleles, minor allele frequency of cases and controls, *p*-value and odds ratio and 95% confidence interval**.

**db SNP ID**	**Chr**	**Position (hg19)**	**Nearby genes**	**Variant location**	**Major/minor alleles**	Case MAF^*^	**control MAF**	**P-value**	**Odds ratio (95% CI)**
rs2455012	1	66520998	*PDE4B*	Intron	C/T	0.090615	0.045190	6.68*E*-07	2.17 (1.59, 2.93)
rs7152530	14	98641215	*VRK1, BCL11B*	Intergenic	G/A	0.270227	0.366746	8.47*E*-07	0.62 (0.51, 0.75)
rs3804047	22	19879637	*TXNRD2*	Intron	T/C	0.368932	0.281143	3.32*E*-06	1.52 (1.27, 1.81)
rs470093	22	44276171	*PNPLA5*	3' UTR	G/T	0.211974	0.146838	7.19*E*-06	1.63 (1.31, 2.02)
rs1381281	14	98615771	*VRK1, BCL11B*	Intergenic	A/G	0.322006	0.410024	1.07*E*-05	0.67 (0.56, 0.80)
rs13107325	4	103188709	*SLC39A8*	Exon 8	G/A	0.113269	0.065299	1.09*E*-05	1.88 (1.41, 2.47)
rs1892234	14	98720904	*VRK1, BCL11B*	Intergenic	T/C	0.364078	0.455897	1.38*E*-05	0.68 (0.57, 0.81)
rs6948646	7	152945284	*ACTR3B, DPP6*	Intergenic	G/A	0.444984	0.362393	1.41*E*-05	1.46 (1.23, 1.73)
rs1012203	9	104626708	*GRIN3A, CYLC2*	Intergenic	A/G	0.048544	0.104103	1.59*E*-05	0.44 (0.29, 0.62)
rs987514	14	98628943	*VRK1, BCL11B*	Intergenic	C/T	0.330097	0.415356	1.95*E*-05	0.68 (0.56, 0.81)
rs11950651	5	103009768	*NUDT12, EFNA5*	Intergenic	C/T	0.032362	0.079829	2.09*E*-05	0.37 (0.22, 0.57)
rs12160908	22	25928620	*LRP5L, ADRBK2*	Intergenic	C/T	0.067961	0.127401	2.13*E*-05	0.49 (0.35, 0.67)
rs12696090	3	158669688	*MFSD1, IQCJ-SCHIP1*	Intergenic	G/T	0.150485	0.226635	2.72*E*-05	0.61 (0.48, 0.76)
rs9614174	22	44082454	*EFCAB6*	Intron	A/C	0.215210	0.151795	2.78*E*-05	1.56 (1.26, 1.92)
rs7255123	19	3958397	*DAPK3*	3' near gene	G/A	0.263754	0.198632	4.11*E*-05	1.51 (1.24, 1.84)
rs6135407	20	15397676	*MACROD2*	Intron	C/T	0.220065	0.158858	4.91*E*-05	1.54 (1.25, 1.90)
rs4807532	19	3928369	*ATCAY*	3' near gene	G/A	0.461165	0.375897	5.32*E*-05	1.42 (1.20, 1.69)
rs7643377	3	142114694	*XRN1*	Intron	C/T	0.483819	0.400342	5.70*E*-05	1.41 (1.19, 1.67)
rs2535368	7	83157207	*SEMA3E*	Intron	G/A	0.202265	0.144494	5.90*E*-05	1.56 (1.25, 1.93)
rs9867210	3	142132749	*XRN1*	Intron	TIC	0.495146	0.411453	6.17*E*-05	1.41 (1.19, 1.67)

* Minor allele frequency.

### VEGAS-based gene analyses

Table 3 shows the top 15 genes ranked by their *p*-values from the VEGAS analysis and four additional interesting genes that could potentially have a role in *S. aureus*-caused diseases based on their known involvement in immune and inflammatory processes. The topmost hit was *NMRK2* (or *ITGB1BP3*; *p* = 1.20E-05) which encodes nicotinamide riboside kinase 2. Two of the 15 genes also featured in the list from the SNP-based analysis: *DAPK3* (*p* = 5.10E-05) and *XRN1* (*p* = 1.85E-04).

**Table 3:** **Top 15 and four other potentially interesting gene hits from VEGAS gene-based analysis**.

**Gene**	**Number of SNPs**	**Chr**.	**Start position (hg19)**	**End position (hg19)**	***p*-value**
*NMRK2 (ITGB1BP3)*	20	19	3933101	3942414	1.20E-05
*DAPK3*	18	19	3958452	3969827	5.10E-05
*NPM3*	4	10	103541082	103543170	0.000142
*EEF2*	17	19	3976054	3985461	0.000167
*XRN1*	12	3	142025449	142166853	0.000185
*CDK7*	*11*	5	68530622	68573257	0.000206
*FBXL4*	17	6	99321601	99395849	0.000257
*ATR*	20	3	142168077	142297668	0.000269
*FGF8*	3	10	103529887	103535759	0.000294
*MRPS36*	8	5	68513573	68525985	0.000423
*CCDC125*	*10*	5	68576519	68616407	0.000424
*ATCAY*	33	19	3880618	3928080	0.000571
*KCNIP2*	6	10	103585731	103603677	0.000637
*MGEA5*	6	10	103544200	103578222	0.000654
*LDOC1L*	30	22	44888450	44894178	0.000664
^*^IL1RL2 (75)	60	2	102803433	102855811	0.0031
^*^IL1B (80)	20	2	113587337	113594356	0.00322
^*^ILIA (93)	22	2	113531492	113542971	0.00363
^*^IL1R1 (168)	54	2	102770401	102796334	0.00806

* Ranks of these genes are mentioned in parenthesis.

### Pathway analyses

The top-ranked 196 genes (*p*-value = 0.01) resulting from the VEGAS analysis were selected for the DAVID analyses but none of the gene groups were statistically significant. One of the top gene groups (gene group 1; Supplemental Table 1) included *CST8* (cystatin 8), *SERPINA6* (serine peptidase inhibitor, clade A, member 6), *SERPINA10* (serine peptidase inhibitor, clade A, member 10), and *SPINK1* (serine peptidase inhibitor, Kazak type 1). The enriched genes in group 2 (Supplemental Table 1) included four keratin genes: *KRT24* (form intermediate filament), *KRT82* (type II hair keratin), *KRT12* (type I intermediate filament keratin 12), and *KRT75* (form intermediate filament in in the cytoplasm of epithelial cells). As expected, the PANTHER analysis also suggested enrichment for intermediate filament cytoskeleton pathway (Supplemental Table 2). Using the same gene set input as in DAVID and PANTHER, the IPA was also explored with the intent of hypothesis-generating protein-protein interaction networks associated our gene data set. The IPA yielded 12 protein networks (data not shown) of which network 7, involving cell death, and survival, appeared interesting (Supplemental Figure 2).

## Discussion

Multiple diseases (endocarditis, skin infections, etc.) caused by *S. aureus* are mediated by two main classes of virulence factors, adhesion and secretory proteins, which interact with host receptors and inflammatory/anti-inflammatory molecules to produce the disease phenotype (Gordon and Lowy, 2008). Adhesion proteins help the pathogen to attach to the skin and survive on the epidermis and in the sub-epidermal layer through a repertoire of molecules collectively known as MSCRAMMS (microbial surface components recognizing adhesive matrix molecules). The MSCRAMMS can bind to fibronectin, fibrinogen, and platelets among others. Subsequent to attachment, *S. aureus* can secrete tissue and organ-specific virulence proteins (e.g., coagulase, proteases, toxins, superantigens) with a wide range of virulence functions that enable the pathogen to infect its host.

So far, most of the genetic susceptibility data related to *S. aureus* has been limited to *S. aureus* colonization. For example, the glucocorticoid receptor gene polymorphisms are associated with carriage risk (van den Akker et al., 2006), whereas *DEFB1* has been shown to promote persistent colonization by modulating beta-defensin expression in keratinocytes (Nurjadi et al., 2013). In contrast, the Danish middle-aged/elderly twin study showed that host genetics had a modest influence only on *S. aureus* carrier state (Andersen et al., 2012). While our manuscript was under review, a study by Nelson et al did not find any SNP that has genome wide significance for association with *S. aureus* bacteremia (SAB) although an intronic SNP in *CDON* was speculated to be associated with complicated SAB (Nelson et al., 2014). In a report by Stappers et al., four SNPs from three toll-like receptor (*TLR*) genes, *TLR1, TLR2*, and *TLR6*, increase the susceptibility to complicated skin and soft tissue infections caused by staphylococci, streptococci, and enterococci (Stappers et al., 2014).

In our multi-tiered GWAS-based investigation, we have identified a number of potentially interesting genes that need further investigation. Although the individual SNP results did not pass genome-wide significance, the top-tier SNPs, Gene-based, and Pathway-based results were enriched for genes that have plausible functions in bacterial infections. This includes genes that have roles in intracellular signaling, inflammation, zinc transport, and integrin binding. Thus, these genes have relatively high prior probabilities for involvement in *S. aureus* infection susceptibility—an aspect of the results that we believe supports considerable interest in subsequent studies to follow-up on these findings.

Notably, two genes (*DAPK3* and *XRN1*) were identified in both the SNP-based and gene-based analyses. *DAPK3* is a protein kinase that modulates apoptosis-related signaling pathways (Wu et al., 2010) and, in interaction with *RhoD*, modulates actin filament assembly and focal adhesion reorganization (Nehru et al., 2013). *S. aureus* is known to induce apoptosis of host cells during host invasion, leading to a compromised host immune response (Haslinger-Löffler et al., 2005). *XRN1* encodes a 5'–3' exonuclease family member involved in cellular mRNA turnover (Nagarajan et al., 2013). The gene is shown to complete host mRNA degradation initiated by viral pathogens (Gaglia et al., 2012). These functions suggest possible roles for *DAPK3* and *XRN1* in susceptibility to diseases caused by *S. aureus*.

Some of the other genes we identified have been implicated in infectious disease processes. *PDE4B* is involved in modulating bacteria-induced inflammation (Komatsu et al., 2013). *PNPLA5* appears to be critical for autophagosome functions, including microbial clearance (Dupont et al., 2014). Mammalian hosts are known to reduce the level of free zinc to thwart pathogen growth (Kehl-Fie and Skaar, 2010) and it is plausible that *SLC39A8*, a zinc transporter, could be involved. *BCL11B* encodes a transcriptional repressor involved in T-cell development (Wakabayashi et al., 2003). *NMRK2*, an integrin beta1 binding protein, could function in host responses to bacterial function based on the finding that host fibronectin forms a bridge between *S. aureus* fibronectin-binding proteins and host cell beta1 integrins during *S. aureus* cellular invasion (Fowler et al., 2000). Keratin intermediate filaments are shown to have a protective role during infection with *Bartonella henselae* in cat scratch disease (Zhu et al., 2013). Interleukin 1 cytokine family members (*IL1A, IL1B, IL1R1*, and *IL1RL2*) are known mediators of immune and inflammatory responses (Garlanda et al., 2013).

It is known that many Mendelian and oligogenetic immunodeficiency disorders confer risk to staphylococcal infection including lymphocyte deficiencies such as severe combined immunodeficiency, chronic granulomatous disease, and hyper-IgE syndrome (Stephan et al., 1993; Grimbacher et al., 1999; Van de Vosse et al., 2009). These disorders are typified by highly disruptive mutations occurring in genes central to lymphoid cell competency including *STAT3, JAK3, DOCK8*, and *CD18*, among others (Hogg et al., 1999; Kalman et al., 2004; Jiao et al., 2008; Zhang et al., 2009). However, most cases of severe staphylococcal infection are not attributable to these more rare conditions and have unknown genetic etiology.

In summary, this preliminary GWAS applied a SNP-to-gene-to-disease-pathway approach to identify susceptibility genes against a broad umbrella of laboratory confirmed *S. aureus* infections. While no one SNP and gene was found to be highly significant in this study, we suspect that for a versatile pathogen like *S. aureus*, that needs to overcome barriers presented by a variety of tissues and defense systems to infect various sites in the body, there are bound to be several genes involved in host susceptibility. Not everyone exposed to a virulent or a colonizing strain of *S. aureus* has similar severity of infection. It is reasonable to speculate that effects of variants segregating at multiple genes contribute to the severity of *S. aureus* infection. Similarly, there could be protective alleles that may lower the risk of clinically-attended infection as well. Additional studies will be needed to confirm these findings but eventually functional studies will be needed to illuminate the detailed mechanisms of how these variants confer predisposition to infection. Once consensus disease loci and pathways are identified, they can serve as targets for future pharmaceutical development and further elucidation of how aberrant cellular processes/signaling give rise to *Staphylococcus*-induced pathologies.

### Conflict of interest statement

The authors declare that the research was conducted in the absence of any commercial or financial relationships that could be construed as a potential conflict of interest.

## Supplementary material

Supplemental Figure 1 **A principal components analysis of genome-wide genotypes in all study subjects**.

Supplemental Figure 2 **One of the IPA outputs of the protein-protein interaction network using VEGAS based 196 genes (p ≤ 0.01) as input**. Three genes, *DAPK3, KRT12*, and *TXNRD2* have been shown to be in direct network UBC (ubiquitin C). Boxes in pink color are input genes from the VEGAS analysis. Network shapes Symbols: ⋄ = enzyme; ∘ = Other; ▽ = Kinase; ^___^ = direct interaction; ----- = indirect interaction. Additional details about the symbols can be found at www.ingenuity.com.

## References

[B1] Andersen P. S. Pedersen J. K. Fode P. Skov R. L. Fowler V. G. Jr. Stegger M. (2012). Influence of host genetics and environment on nasal carriage of *Staphylococcus aureus* in Danish middle-aged and elderly twins. J. Infect. Dis. 206, 1178–1184 10.1093/infdis/jis491 22872733 PMC3448969

[B2] Burton P. R. Clayton D. G. Cardon L. R. Craddock N. Deloukas P. Duncanson A. (2007). Genome-wide association study of 14,000 cases of seven common diseases and 3,000 shared controls. Nature 447, 661–678 10.1038/nature05911 17554300 PMC2719288

[B3] Cross D. S. McCarty C. A. Hytopoulos E. Beggs M. Nolan N. Harrington D. (2012). Coronary risk assessment among intermediate risk patients using a clinical and biomarker based algorithm developed and validated in two population cohorts. Curr. Med. Res. Opin. 28, 1819–1830 10.1185/03007995 23092312 PMC3666558

[B4] de Bakker P. I. Telenti A. (2010). Infectious diseases not immune to genome-wide association. Nat. Genet. 42, 731–732 10.1038/ng0910-731 20802473

[B5] Dupont N. Chauhan S. Arko-Mensah J. Castillo E. F. Masedunskas A. Weigert R. (2014). Neutral lipid stores and lipase PNPLA5 contribute to autophagosome biogenesis. Curr. Biol. 24, 609–620 10.1016/j.cub.2014.02.008 24613307 PMC4016984

[B6] Fowler T. Wann E. R. Joh D. Johansson S. Foster T. J. Höök M. (2000). Cellular invasion by *Staphylococcus aureus* involves a fibronectin bridge between the bacterial fibronectin-binding MSCRAMMs and host cell beta1 integrins. Eur. J. Cell Biol. 79, 672–679 10.1078/0171-9335-00104 11089915

[B7] Gaglia M. M. Covarrubias S. Wong W. Glaunsinger B. A. (2012). A common strategy for host RNA degradation by divergent viruses. J. Virol. 86, 9527–9530 10.1128/JVI.01230-12 22740404 PMC3416159

[B8] Garlanda C. Dinarello C. A. Mantovani A. (2013). The interleukin-1 family: back to the future. Immunity 39, 1003–1018 10.1016/j.immuni.2013.11.010 24332029 PMC3933951

[B9] Gordon R. J. Lowy F. D. (2008). Pathogenesis of methicillin-resistant *Staphylococcus aureus* infection. Clin. Infect. Dis. 46 Suppl. 5, S350–S359 10.1086/533591 18462090 PMC2474459

[B10] Graffunder E. M. Venezia R. A. (2002). Risk factors associated with nosocomial methicillin-resistant *Staphylococcus aureus* (MRSA) infection including previous use of antimicrobials. J. Antimicrob. Chemother. 49, 999–1005 10.1093/jac/dkf009 12039892

[B11] Graham P. L. 3rd. Lin S. X. Larson E. L. (2006). A. US population based survey of Staphylococcus aureus colonization. Ann. Intern. Med. 144, 318–325 10.7326/0003-4819-144-5-200603070-00006 16520472

[B12] Grimbacher B. Holland S. M. Gallin J. I. Greenberg F. Hill S. C. Malech H. L. (1999). Hyper-IgE syndrome with recurrent infections—an autosomal dominant multisystem disorder. N. Engl. J. Med. 340, 692–702 10.1056/NEJM199903043400904 10053178

[B13] Haslinger-Löffler B. Kahl B. C. Grundmeier M. Strangfeld K. Wagner B. Fischer U. (2005). Multiple virulence factors are required for *Staphylococcus aureus*-induced apoptosis in endothelial cells. Cell. Microbiol. 7, 1087–1097 10.1111/j.1462-5822.2005.00533.x 16008576

[B14] Hebbring S. Slager S. Epperla N. Mazza J. J. Ye Z. Zhou Z. (2013). Genetic Evidence of *PTPN22* effects on chronic lymphocytic leukemia. Blood 121, 237–238 10.1182/blood-2012-08-450221 23287625 PMC5091216

[B15] Hogg N. Stewart M. P. Scarth S. L. Newton R. Shaw J. M. Law S. K. (1999). A novel leukocyte adhesion deficiency caused by expression but nonfunctional beta-2 integrins Mac-1 and LFA-1. J. Clin. Invest. 103, 97–106 10.1172/JCI3312 9884339 PMC407855

[B16] Huang D. A. W. Sherman B. T. Lempicki R. A. (2009a). Systematic and integrative analysis of large gene lists using DAVID Bioinformatics Resources. Nat. Protoc. 4, 44–57 10.1038/nprot.2008.211 19131956

[B17] Huang D. A. W. Sherman B. T. Lempicki R. A. (2009b). Bioinformatics enrichment tools: paths toward the comprehensive functional analysis of large gene lists. Nucleic Acids Res. 37, 1–13 10.1093/nar/gkn923 19033363 PMC2615629

[B18] Huang D. A. W. Sherman B. T. Tan Q. Collins J. R. Alvord W. G. Roayaei J. (2007). The DAVID gene functional classification tool: a novel biological module-centric algorithm to functionally analyze large gene lists. Genome Biol. 8:R183 10.1186/gb-2007-8-9-r183 17784955 PMC2375021

[B19] Jiao H. Tóth B. Erdos M. Fransson I. Rákóczi E. Balogh I. (2008). Novel and recurrent STAT3 mutations in hyper-IgE syndrome patients from different ethnic groups. Mol. Immunol. 46, 202–206 10.1016/j.molimm.2008.07.001 18706697

[B20] Kalman L. Lindegren M. L. Kobrynski L. Vogt R. Hannon H. Howard J. T. (2004). Mutations in genes required for T-cell development: IL7R, CD45, IL2RG, JAK3, RAG1, RAG2, ARTEMIS, and ADA and severe combined immunodeficiency: HuGE review. Genet. Med. 6, 16–26 10.1097/01.GIM.0000105752.80592.A3 14726805

[B21] Kehl-Fie T. E. Skaar E. P. (2010). Nutritional immunity beyond iron: a role for manganese and zinc. Curr. Opin. Chem. Biol. 14, 218–224 10.1016/j.cbpa.2009.11.008 20015678 PMC2847644

[B22] Komatsu K. Lee J. Y. Miyata M. Hyang Lim J. Jono H. Koga T. (2013). Inhibition of PDE4B suppresses inflammation by increasing expression of the deubiquitinase CYLD. Nat. Commun. 4, 1684 10.1038/ncomms2674 23575688 PMC3644066

[B23] Kuehnert M. J. Kruszon-Moran D. Hill H. A. McQuillan G. McAllister S. K. Fosheim G. (2006). Prevalence of *Staphylococcus aureus* nasal colonization in the United States, 2001-2002. J. Infect. Disease. 193, 169–171 10.1086/499632 16362880

[B24] Liu J. Z. McRae A. F. Nyholt D. R. Medland S. E. Wray N. R. Brown K. M. (2010). A versatile gene-based test for genome-wide association studies. Am. J. Hum. Genet. 87, 139–145 10.1016/j.ajhg.2010.06.009 20598278 PMC2896770

[B25] Lowy F. D. (1998). *Staphylococcus aureus* infections. N. Engl. J. Med. 339, 520–532 10.1056/NEJM199808203390806 9709046

[B26] McCarty C. A. Chisholm R. L. Chute C. G. Kulo I. J. Jarvi G. P. Larson E. B. (2011). The eMERGE Network: a consortium of biorepositories linked to electronic medical records data for conducting genomic studies. BMC Med. Genomics 4:13 10.1186/1755-8794-4-13 21269473 PMC3038887

[B27] Mi H. Thomas P. (2009). PANTHER pathway: an ontology-based pathway database coupled with data analysis tools. Methods Mol. Biol. 563, 123–140 10.1007/978-1-60761-175-2_7 19597783 PMC6608593

[B28] Nagarajan V. K. Jones C. I. Newbury S. F. Green P. J. (2013). *XRN* 5'?3' exoribonucleases: structure, mechanisms and functions. Biochim. Biophys. Acta 1829, 590–603 10.1016/j.bbegrm.2013.03.005 23517755 PMC3742305

[B29] Neale B. M. Sham P. C. (2004). The future of association studies: gene-based analysis and replication. Am. J. Hum. Genet. 75, 353–362 10.1086/423901 15272419 PMC1182015

[B30] Nehru V. Almeida F. N. Aspenström P. (2013). Interaction of RhoD and ZIP kinase modulates actin filament assembly and focal adhesion dynamics. Biochem. Biophys. Res. Commun. 433, 163–169 10.1016/j.bbrc.2013.02.046 23454120

[B31] Nelson C. L. Pelak K. Podgoreanu M. V. Ahn S. H. Scott W. K. Allen A. S. (2014). A genome-wide association study of variants associated with acquisition of *Staphylococcus aureus* bacteremia in a healthcare setting. BMC Infect. Dis. 14:83 10.1186/1471-2334-14-83 24524581 PMC3928605

[B32] Nurjadi D. Herrmann E. Hinderberger I. Zanger P. (2013). Impaired β-defensin expression in human skin links DEFB1 promoter polymorphisms with persistent *Staphylococcus aureus* nasal carriage. J. Infect. Dis. 207, 666–674 10.1093/infdis/jis735 23204181

[B33] Price A. L. Patterson N. J. Plenge R. M. Weinblatt M. E. Shadick N. A. Reich D. (2006). Principal components analysis corrects for stratification in genome-wide association studies. Nat. Genet. 38, 904–909 10.1038/ng1847 16862161

[B34] Purcell S. Neale B. Todd-Brown K. Thomas L. Ferreira M. A. R. Bender D. (2007). PLINK: a toolset for whole-genome association and population-based linkage analysis. Am. J. Hum. Genet. 81, 559–575 10.1086/519795 17701901 PMC1950838

[B35] R. Core Team., (2013). R: a language and environment for statistical computing, in R Foundation for Statistical Computing, Vienna, Austria. Available online at: http://www.R-project.org/

[B36] Rehm S. J. (2008). *Staphylococcus aureus*: the new adventures of a legendary pathogen. Cleve. Clin. J. Med. 75, 177–180, 183–186, 190–192. 10.3949/ccjm.75.3.177 18383927

[B37] Stappers M. H. Thys Y. Oosting M. Plantinga T. S. Ioana M. Reimnitz P. (2014). TLR1, TLR2, and TLR6 gene polymorphisms are associated with increased susceptibility to complicated skin and skin structure infections. J. Infect. Dis. [Epub ahead of print]. 10.1093/infdis/jiu080 24511099

[B38] Stephan J. L. Vlekova V. Le Deist F. Blanche S. Donadieu J. De Saint-Basile G. (1993). Severe combined immunodeficiency: a retrospective single-center study of clinical presentation and outcome in 117 patients. J. Pediatr. 123, 564–572 10.1016/S0022-3476(05)80951-5 8410508

[B39] Van de Vosse E. van Wengen A. van Geelen J. A. de Boer M. Roos D. van Dissel J. T. (2009). A novel mutation in NCF1 in an adult CGD patient with a liver abscess as first presentation. J. Hum. Genet. 54, 313–316 10.1038/jhg.2009.24 19329991

[B40] van den Akker E. L. Nouwen J. L. Melles D. C. van Rossum E. F. Koper J. W. Uitterlinden A. G. (2006). *Staphylococcus aureus* nasal carriage is associated with glucocorticoid receptor gene polymorphisms. J. Infect. Dis. 194, 814–818 10.1086/506367 16941349

[B41] Wakabayashi Y. Watanabe H. Inoue J. Takeda N. Sakata J. Mishima Y. (2003). Bcl11b is required for differentiation and survival of alphabeta T lymphocytes. Nat. Immunol. 4, 533–539 10.1038/ni927 12717433

[B42] Wu Y. Yan Q. Zuo J. Saiyin H. Jiang W. Qiao S. (2010). Link of Dlk/ZIP kinase to cell apoptosis and tumor suppression. Biochem. Biophys. Res. Commun. 392, 510–515 10.1016/j.bbrc.2010.01.054 20085750

[B43] Yang J. Manolio T. A. Pasquale L. R. Boerwinkle E. Caporaso N. Cunningham J. M. (2011). Genome partitioning of genetic variation for complex traits using common SNPs. Nat. Genet. 43, 519–525 10.1038/ng.823 21552263 PMC4295936

[B44] Zhang Q. Davis J. C. Lamborn I. T. Freeman A. F. Jing H. Favreau A. J. (2009). Combined immunodeficiency associated with DOCK8 mutations. N. Engl. J. Med. 361, 2046–2055 10.1056/NEJMoa0905506 19776401 PMC2965730

[B45] Zhu C. Bai Y. Liu Q. Li D. Hong J. Yang Z. (2013). Depolymerization of cytokeratin intermediate filaments facilitates intracellular infection of HeLa cells by Bartonella henselae. J. Infect. Dis. 207, 1397–1405 10.1093/infdis/jit040 23359593

