# Supplementary figures and images for: Genome wide association study of SNP-, gene-, and pathway-based approaches to identify genes influencing susceptibility to Staphylococcus aureus infections
Source: Front Genet. 2014 May 9;5:125. doi: 10.3389/fgene.2014.00125 (PMC4023021; doi:10.3389/fgene.2014.00125)

**Eigenvectors 1 and 2 from Cases and controls**

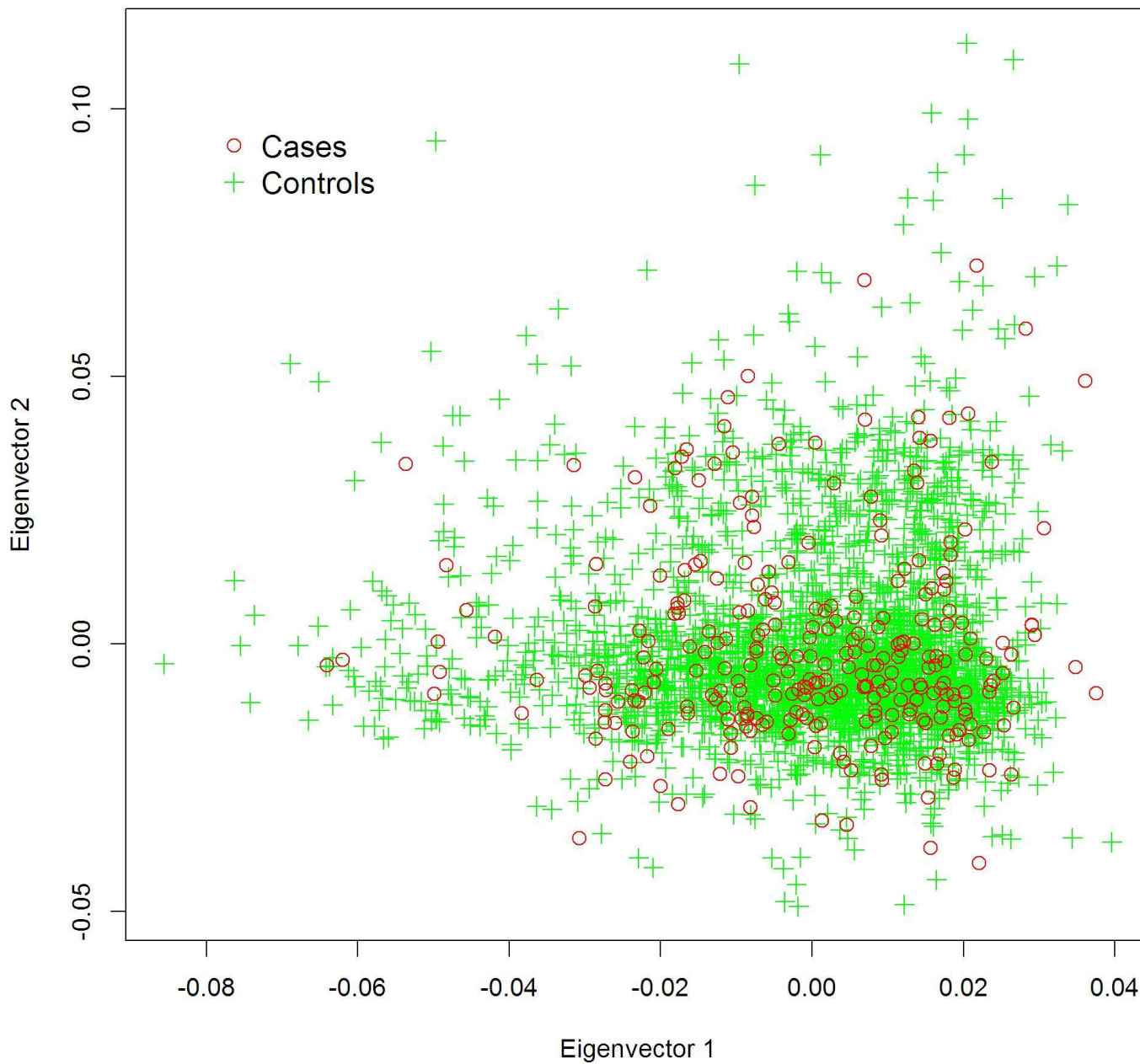

Supplement: Supplemental Figure 1 — A principal components analysis of genome-wide genotypes in all study subjects. [file Presentation1.PDF]

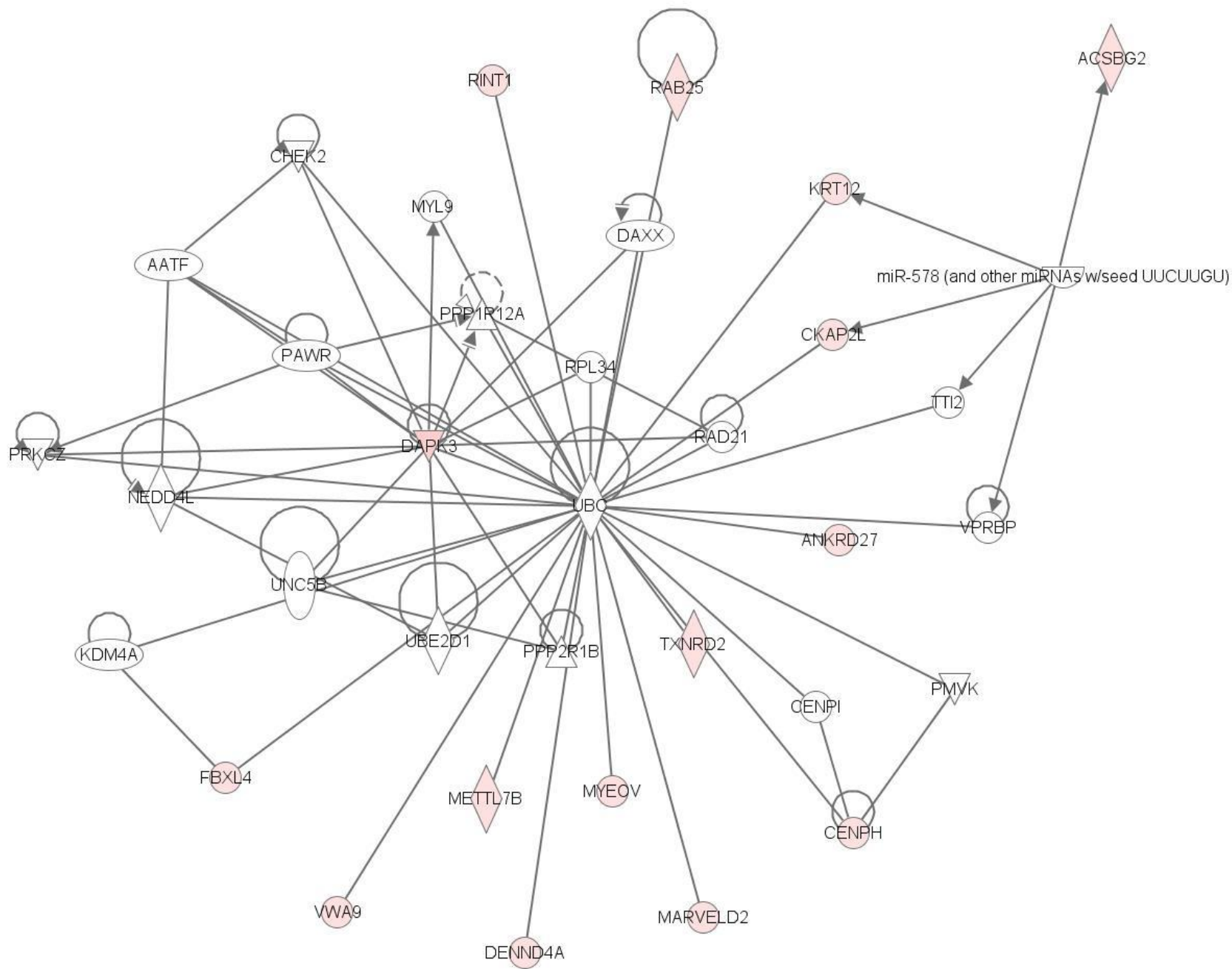

Supplement: Supplemental Figure 2 — One of the IPA outputs of the protein-protein interaction network using VEGAS based 196 genes (p ≤ 0.01) as input. Three genes, DAPK3, KRT12, and TXNRD2 have been shown to be in direct network UBC (ubiquitin C). Boxes in pink color are input genes from the VEGAS analysis. Network shapes Symbols: ⋄ = enzyme; ∘ = Other; ▽ = Kinase; ___ = direct interaction; ----- = indirect interaction. Additional details about the symbols can be found at www.ingenuity.com. [file Presentation2.PDF]
