# Supplementary material for: Genome wide association study of SNP-, gene-, and pathway-based approaches to identify genes influencing susceptibility to Staphylococcus aureus infections
Source: Front Genet. 2014 May 9;5:125. doi: 10.3389/fgene.2014.00125 (PMC4023021; doi:10.3389/fgene.2014.00125)
Supplement: Supplementary file 3 [file DataSheet1.PDF]

Supplemental Table 1. The DAVID analysis yielded two gene groups, Gene Group 1 and 2 along with the enrichment score. *The enrichment score is calculated using gene-term enrichment algorithms (see Huang et al. 2007). The enrichment score of the each group is measured by the geometric mean of the EASE (Expression Analysis Systemic Explorer) score (see Hosack et al. 2003) and is negative logarithm transformation of the p-value from the modified Fisher exact test.*

Hosack DA, Dennis G Jr, Sherman BT, Lane HC and Lempicki RA. Identifying biological themes within lists of genes with EASE. Genome Biol. 2003;4(10):R70.

Gene Group 1	***Enrichment Score: 0.93***
CST8:	cystatin 8 (cystatin-related epididymal specific)
SERPINA6:	serpin peptidase inhibitor, clade A (alpha-1 antiproteinase, antitrypsin), member 6
SPINK1:	serine peptidase inhibitor, Kazal type 1
SERPINA10:	serpin peptidase inhibitor, clade A (alpha-1 antiproteinase, antitrypsin), member 10

Gene Group 2	***Enrichment Score: 0.91***
KRT24:	keratin 24
KRT82:	keratin 82
KRT12:	keratin 12
KRT75:	keratin 75
