# Supplementary material for: Genome wide association study of SNP-, gene-, and pathway-based approaches to identify genes influencing susceptibility to Staphylococcus aureus infections
Source: Front Genet. 2014 May 9;5:125. doi: 10.3389/fgene.2014.00125 (PMC4023021; doi:10.3389/fgene.2014.00125)
Supplement: Supplementary file 4 [file DataSheet2.PDF]

Supplemental Table 2. Results from the PANTHER program.

Cellular Component	*Homo sapiens REFLIST (21804)*	*Input Gene list (195)*	*Input Gene list (expected)*	*Input Gene list (over/under)*	*Input Gene list (P-value)*	*Bonferroni Correction*
intermediate filament cytoskeleton	73	5	0.65	+	2.88E-04	2.83E-02
